# Diets with Higher Vegetable Intake and Lower Environmental Impact: Evidence from a Large Australian Population Health Survey

**DOI:** 10.3390/nu14071517

**Published:** 2022-04-05

**Authors:** Bradley Ridoutt, Danielle Baird, Gilly A. Hendrie

**Affiliations:** 1 Commonwealth Scientific and Industrial Research Organisation (CSIRO) Agriculture and Food, Clayton, VIC 3169, Australia; 2 Department of Agricultural Economics, University of the Free State, Bloemfontein 9300, South Africa; 3 CSIRO Health and Biosecurity, Adelaide, SA 5000, Australia; danielle.baird@csiro.au (D.B.); gilly.hendrie@csiro.au (G.A.H.)

**Keywords:** climate change, cropland footprint, dietary guidelines, diet quality, environmental footprint, life-cycle assessment, pesticide footprint, sustainable diet, variety, water footprint

## Abstract

Increasing the consumption of vegetables is a public health nutrition priority in Australia. This must be achieved in the context of lowering dietary environmental impacts. In this study, a subgroup of 1700 Australian adult daily diets having a higher diet-quality score and a lower environmental impact score was isolated from Australian Health Survey data. These diets were primarily distinguished by their lower content of energy-dense/nutrient-poor discretionary foods. Among these diets, those with higher levels of vegetable intake were characterized by greater variety of vegetables eaten, lower intake of bread and cereal foods, and higher intake of red meat. These diets also had a greater likelihood of achieving recommended intakes for a range of vitamins and minerals. These findings highlighted the importance of considering the total diet in developing strategies to promote healthy and sustainable food consumption, as well as the need to understand the interrelationships between foods that exist in a local cultural context. As vegetables are usually eaten with other foods, higher vegetable consumption in Australia could be supported by encouraging more regular consumption of the types of meals that include larger quantities of vegetables. Our results showed that this was possible while also substantially lowering total dietary environmental impacts.

## 1. Introduction

In Australia, fewer than 10% of adults meet the daily recommended intake of vegetables [1], and there is little evidence of substantial change over time [2]. This represents a major public health nutrition concern, as higher levels of fruit and vegetable intake are associated with many health and wellbeing benefits, and inadequate levels of fruit and vegetable intake are linked to poor health and higher disease risk [3]. For example, higher levels of fruit and vegetable intake are linked to greater life expectancy [4,5,6], as well as a reduced risk of cardiovascular disease, cancer [7,8,9], and inflammation [10], including asthma [11]. Higher levels of fruit and vegetable intake may also help to address problems of overweight and obesity [12,13]. The potential benefits of higher fruit and vegetable consumption are diverse, including reduced incidence of headaches [14], maintenance of telomere length [15], and improved mental health [16,17,18,19]. Higher fruit and vegetable intake has also been associated with a range of other healthy lifestyle factors, including higher rates of exercise and not smoking [20,21]. Not surprisingly, there is also evidence that healthcare costs are lower for adults who consume more fruits and vegetables [22]. Based on these many associations, there is a more-or-less universal agreement among scientists, policymakers, and health practitioners of the need to support higher levels of fruit and vegetable consumption in the community. However, in Australia, the situation is far more acute for vegetables than for fruits. While Australian adults are encouraged to consume more whole fruit [23], their total intake of fruits from all sources, including fruits contained in processed foods, desserts, and fruit-based beverages, approximates the *Australian Dietary Guidelines’* recommended 350 g per capita per day [2]. In contrast, vegetables are the most underconsumed food group across the Australian adult population [24].

In addition to encouraging the adoption of more healthy diets containing a higher intake of vegetables, there is also the need to work toward more sustainable dietary habits that have lower environmental impacts. At the global level, the environmental impacts of food production are broadly viewed as unsustainable, and there is a need to address the levels of greenhouse gas emissions, water consumption, and other impacts related to land use and the application of fertilizers and pesticides during agricultural production [25,26,27]. On the one hand, improving sustainability across the food system requires innovation in the methods of food production, processing, and distribution. However, changing dietary habits could also play a role [28,29,30,31,32,33]. Regarding the latter, which is more within the domain of dietitians and healthcare practitioners, one recommendation is to limit the intake of livestock products, especially ruminant livestock products such as beef, lamb, and goat meat, along with dairy foods [34,35,36,37,38]. However, this approach is not without its critics, as the health risks of discouraging the intake of nutrient-dense, animal-sourced foods has been raised, especially in regions such as Australia, where these foods have traditionally formed part of the diet and are an important source of nutrients that are widely underconsumed across the population [39,40,41]. There is also evidence that many diets with lower greenhouse gas emissions have poor nutritional and health indicators [42,43,44]. Other approaches to reducing dietary environmental impacts have focused on a reduction in food waste [45,46] and the limitation of discretionary foods and beverages that are high in dietary energy but contribute few nutrients [47,48,49]. These indulgence foods inflate dietary environmental impacts and predominantly have negative health implications, contributing to excessive dietary energy intake and displacing the adequate intake of healthy core foods, including vegetables [50,51].

In previous research, a life-cycle assessment (LCA) was used to characterize the environmental impacts for 9341 individual Australian adult daily diets obtained from the Australian Health Survey [52,53,54,55]. Using this data resource, we examined a subgroup of Australian adult diets characterized by a higher diet-quality score and a lower environmental impact score. This subgroup is important because it represents the food habits of Australian adults that have more desirable characteristics and are realistic, since they are already prevalent within the community. Our goal was to assess vegetable intake within this subgroup, and to characterize the food choices related to higher vegetable intake, as well as the implications for nutritional adequacy. Our purpose was to support the development of strategies for higher vegetable consumption in Australia that are consistent with the wider objectives of nutritional adequacy and sustainability. The study was novel in its assessment of vegetable intake in the context of complete diets with a lower environmental impact.

## 2. Materials and Methods

### 2.1. Background Data

Dietary intake data for 9341 Australian adults (19 years old and above) were obtained from the nutrition component [24] of the Australian Health Survey [56]. These data were collected by the Australian Bureau of Statistics using a 24-h recall process over a 13-month period and across all days of the week to capture variation in eating habits across seasons and including weekends. The survey employed a complex sampling methodology to enable the estimation of dietary intake for the Australian population, as well as demographic subgroups, through the application of population-weighting factors. As such, the dataset is nationally representative and comprehensive in that intake was recorded for more than 5000 food and beverage categories. Nevertheless, as with any 24-h dietary-recall process, there is potential for inaccurate recall of foods and portion sizes. To address this issue, the Australian Bureau of Statistics has also published estimates of the prevalence of under-reporting of energy (17% for males and 21% for females) [56] that were used in this study to uniformly adjust the dietary intake data to avoid systematic underestimation of environmental impacts, and to enable reliable comparison with recommended daily intakes described in the Australian Dietary Guidelines [23]. To facilitate analysis of food patterns, composite foods and mixed dishes were disaggregated into basic uncooked components, as explained in previous studies [52,53,54]. Food classification into food groups was undertaken following the *Australian Dietary Guidelines* [23], avoiding subjective decisions about the way foods were grouped. At the highest level, the *Australian Dietary Guidelines* identified five core food groups: fruits; vegetables; grain (cereal) foods; fresh meats and other protein-rich foods such as tofu, nuts, and eggs; and finally dairy foods, which include dairy alternatives made from soy or nuts, etc. In addition, the *Australian Dietary Guidelines* describe “discretionary choices”, which are energy-dense and nutrient-poor foods and beverages high in saturated fats, added sugars, and salt, and includes alcoholic beverages. This covers many types of foods widely eaten in Australia, such as cakes and biscuits, ice cream and other dairy desserts, processed meats, potato chips, confectionaries, extruded snacks, conserves, and the like.

Environmental data for individual foods within the Australian food system were obtained from previous studies. These data, derived from life-cycle assessments, included the water-scarcity footprint [57], climate footprint [54], cropland-scarcity footprint [53], and pesticide-toxicity footprint [55].

### 2.2. Higher Diet Quality and Lower Environmental Impact Subgroup

A subset of adult daily diets was identified with the characteristics of a higher diet-quality score and a lower environmental impact score. Diet quality was assessed using the Diet Quality Index created by Golley and Hendrie [58]. This index describes the level of compliance with the food-based *Australian Dietary Guidelines* [23]. The scores range from 0 to 100, where a higher score reflects greater compliance with the guidelines. The dietary environmental impact was assessed using an adaptation of the environmental impact score presented by Ridoutt et al. [59]. The original environmental impact score [59] included three environmental aspects, namely climate impact, water-scarcity impact, and cropland-scarcity impact, based on data available at that time for individual foods in the Australian food system. The environmental impact score used in this study also integrated pesticide-toxicity data that became recently available [55] using weighting factors presented in Supplementary Table S1. Details of the method used to combine the different environmental indicators into a combined score are presented in the associated reference [59]. In brief, weighting factors were developed using a distance-to-target method in which the relative distance to target determined the weight [60]. Environmental indicators requiring a large improvement were given a larger weight compared to indicators that required less improvement.

The 9341 individual daily diets were subsequently sorted into four quadrants according to diet-quality score and environmental impact score, stratified by gender and the Nutrient Reference Value age group (i.e., 19–30, 31–50, 51–70, and 70 years and above) [61]. The stratification process was necessary, as it was previously established that environmental impacts were positively correlated with total energy intake [52,53,54,62]. This process made it possible to achieve a balance of daily diets in each quadrant according to gender and age group. Without this process, the higher diet quality and lower environmental impact subgroup would have been biased toward females and those of older age who, on average, consumed less dietary energy. To provide a greater contrast, daily diets within a 0.25 standard deviation of the mean of each parameter were excluded. Through these steps, a subset of 1700 daily diets with a higher diet-quality score and a lower environmental-impact score (HQLI diets) were isolated for further analysis. Supplementary Table S2 describes the composition by age group and gender.

### 2.3. Analysis of Vegetable Intake

For each of the 1700 HQLI daily diets, the quantity of vegetables was assessed in servings and grams, excluding vegetables classified as discretionary choices (e.g., fried potato chips and crisps). Following the *Australian Dietary Guidelines* [23], one serving of vegetables referred to a 75 g portion or one cup of green leafy or raw salad vegetables. In addition, the variety of vegetables in each of the 1700 HQLI daily diets was also assessed. The Australian Health Survey groups vegetables into 10 subcategories: potatoes; cabbage, cauliflower, and brassica; carrot and root; leaf and stalk; peas and beans; tomato and tomato products; other fruiting vegetables; other vegetables and combinations; legumes and pulses; and dishes with vegetables. A variety score (out of 10) was calculated for each daily diet based on the number of subcategories consumed, with no minimum level of intake necessary. For the sensitivity analysis, an alternative scoring method was applied whereby only intake greater than 0.25 serving counted toward the variety score. Here, it is important to note that the *Australian Dietary Guidelines* classify potato or other starchy vegetables (sweet potato, taro, or cassava) within the “Vegetable” food group [23]. However, fried potato crisps or chips are not included.

The 1700 HQLI daily diets were subsequently divided into tertiles based on vegetable intake, and were separately assessed in terms of food and nutrient intake. Two additional groups were also formed based on whether the *Australian Dietary Guidelines’* [23] recommendations for vegetable intake were met or not. According to the *Australian Dietary Guidelines*, adult women should eat a minimum of 5 servings of vegetables per day. For men, the recommendation is a minimum of 6 servings per day for the ages of 19 to 50, 5.5 servings per day for the ages of 51 to 70, and 5 servings per day for ages older than 70.

### 2.4. Nutrient Profiling

Nutrient profiling was undertaken for 32 macro- and micronutrients in relation to the Nutrient Reference Values published by the National Health and Medical Research Council in Australia [61]. Data describing the nutrient content of foods and beverages in the Australian food system were obtained from the Australian Food Composition Database [63]. Each of the 1700 adult daily diets in the HQLI subgroup was characterized. These data were used to assess the nutrient density (i.e., per MJ dietary energy) and whether estimated average requirements (EARs) were met [61].

### 2.5. Statistical Analyses

Statistical analyses were performed using IBM SPSS statistical software package version 26. Summary estimates were weighted to reflect the demographic structure of the Australian population, with an additional weighting factor applied to correct for the day of the week on which the survey was recorded. To test for differences in baseline characteristics between the HQLI subgroup and population estimates, a one-sample t-test for continuous variables and a chi-squared test for goodness of fit for categorical variables were used. The HQLI subgroup was removed from the population sample prior to running these tests (*n* = 7641). A one-sample t-test was also used to test for differences in the nutrient density of the HQLI subgroup compared to the population estimate. The Pearson correlation coefficient was used to evaluate the relationship between the dietary energy intake and the environmental impact scores, as well as between the reported vegetable intake and the variety of vegetables consumed (*n* = 1700).

## 3. Results

### 3.1. Characteristics of the HQLI Subgroup

Compared to the average Australian adult daily diet, the HQLI subgroup had a 39% higher diet-quality score (59.2 compared to 42.6; Table 1) and a 41% lower environmental-impact score (0.059 compared to 0.101). Importantly, the HQLI subgroup of diets had lower environmental impacts across all of the four indicators assessed (Table 1). For example, the climate footprints of these diets were less than half that of the average Australian daily diet (1.55 kg CO_2_e compared to 3.33 kg CO_2_e; Table 1). The main factor differentiating the HQLI subgroup of diets was a lower intake of discretionary choices (2.3 servings per day compared to 6.8 for the average adult diet; Supplementary Table S3). Vegetable consumption was also 30% higher. Differences in intake for other food groups were much smaller, and there was no significant difference in overall consumption of meat and alternatives. The HQLI subgroup of diets was also associated with Australian adults who were more active and less likely to smoke cigarettes. However, their weight status was not found to be significantly different. Adults within the HQLI subgroup were also more likely to have higher levels of formal education. However, differences in socio-economic status were not evident (Table 1).

The HQLI subgroup of daily diets was identified by a quadrant analysis using a diet-quality index that assessed the overall compliance with the *Australian Dietary Guidelines* [23]. In accordance with the higher intake of core foods and lower intake of energy-dense and nutrient-poor discretionary choices, these daily diets had a substantially lower total energy intake compared to the population average (7945 kJ compared to 10,458 kJ) and a higher nutrient density (Table 2). The nutrient density was more than 20% higher for long-chain omega-3 polyunsaturated fatty acids, retinol equivalents, dietary fiber and dietary folate, and more than 10% higher for vitamins B1, B2, B3, C, and E, as well as magnesium, potassium, selenium, iron, iodine, and calcium. In addition, there were lower amounts of sodium, free sugars, trans-fatty acids, and alcohol.

### 3.2. Vegetable Intake and Variety

Daily diets were selected for the HQLI subgroup on the basis of having a higher overall diet-quality score. As such, there was substantial variation in vegetable intake within the group. Within the HQLI subgroup, the lowest vegetable intake tertile had an intake of only 0.29 serving (Table 3), which could be considered very low, as Australian recommendations for adults vary from 5 to 6 servings per day. These diets also had the least variety in types of vegetables consumed. In contrast, more than 7.5 servings of vegetables were included in the diets of those in the highest vegetable-intake tertile, and the variety of vegetables consumed was highest. The total quantity of vegetables consumed and the variety of vegetable consumption were positively correlated (r = 0.3 to 0.6 depending on variety index, *p* < 0.01; *n* = 1700). Among the 1700 HQLI daily diets, 24% met the *Australian Dietary Guidelines’* recommended intake of vegetables. These diets also included almost nine servings of vegetables each per day, and had the greatest variety (Table 3).

### 3.3. Dietary Patterns

Two main factors differentiated HQLI daily diets with higher and lower vegetable intakes. Firstly, HQLI diets with a higher vegetable intake also had a lower intake of bread and cereal foods (4.32 servings compared to 5.60 servings; *p* < 0.001; Table 4). Secondly, diets with higher vegetable intake also had higher red meat intake, both beef and lamb (0.69 serving compared to 0.28 serving; *p* < 0.001; Table 4) and pork (0.19 serving compared to 0.08 serving; *p* = 0.002; Table 4). The intake of other protein-rich foods, such as fish, poultry, and vegetarian alternatives, did not differ significantly. Likewise, the intake of fruits was not found to differ. There were differences in discretionary choices between the higher and lower vegetable intake tertiles (2.55 compared to 2.15 servings; Table 4); however, these levels of discretionary food intake were both well below the population estimate of 6.8 servings (Supplementary Table S3), and within the *Australian Dietary Guidelines’* recommended amounts. There were also smaller differences in the intake of dairy foods and alternatives, and in offals and reptiles. HQLI daily diets that met the *Australian Dietary Guidelines’* recommendations for vegetable intake also had a lower intake of bread and cereal foods and a higher intake of red meat compared to diets not meeting the recommendation for vegetables (Table 4). In addition, there was also a difference in discretionary food intake (2.19 compared to 2.70 servings; Table 4), which, as noted above, was well below the population estimate of 6.8 servings (Supplementary Table S3) and within the *Australian Dietary Guidelines’* recommended amounts. A smaller difference in poultry consumption was also observed (Table 4). A further observation was that HQLI daily diets with a lower vegetable intake contained more than half (51.8%) of their vegetables as mixed dishes; for example, in a casserole, in which vegetables were incorporated into the recipe. In contrast, for HQLI diets with a higher vegetable intake, only 18.7% of vegetable intake was through mixed dishes, and the majority were from other various categories.

### 3.4. Nutrient Adequacy

HQLI diets with a higher intake of vegetables were also more likely to achieve the recommended levels of intake of a variety of vitamins and minerals. The largest difference was for vitamin A (retinol equivalents), for which 76% of daily diets in the high vegetable intake tertile met the EAR, compared to only 38.6% in the low vegetable intake tertile (Table 5). Other large differences (>20%) were observed for vitamins B6 and C, as well as for magnesium and zinc. Smaller differences (>10%) were observed for vitamin B1 and iron. Similar results were also evident when comparing HQLI diets that met the *Australian Dietary Guidelines’* recommendations for vegetable intake compared to those that did not (Table 5). The differences in nutrient adequacy of daily diets was partly explained by the differences in vegetable intake, but also partly explained by the differences in food intake reported in Section 3.3 (Table 4). For example, the higher levels of intake of red meat associated with a higher vegetable intake contributed to meeting the EAR for zinc.

## 4. Discussion

### 4.1. Higher-Quality Diets with Lower Environmental Impacts

This was the first study in Australia to assess diets with a higher vegetable intake within the context of sustainability; specifically, environmental sustainability. The study design first involved the isolation of a higher diet quality and lower environmental impact (HQLI) subgroup of Australian adult daily diets. This subgroup of diets was important, as it reflected the actual dietary habits of Australians who already have more desirable dietary characteristics. As these diets are prevalent in the community, they can be considered to be realistically able to be adopted by Australians whose diets are presently of poorer quality and/or have higher environmental impacts. These daily diets also reflect combinations of foods that make sense in the local cultural context. Most foods are not consumed independently, but are grouped together to form meals. The HQLI subgroup of diets can therefore be regarded as culturally acceptable, which is a foundational principle of sustainable diets [64]. It is also important to underscore that the HQLI subgroup was identified by a quadrant analysis from within the observed dietary intake data obtained from the Australian Health Survey. This approach is preferred to the use of hypothetical dietary scenarios, which are subjectively defined and susceptible to researcher biases. Whereas studies based on hypothetical dietary scenarios tend to involve the exclusion of particular foods and food groups [28], the Australian evidence was that higher quality diets with lower environmental impacts were diverse, and that they were primarily distinguished by a lower intake of energy-dense and nutrient-poor discretionary foods (Supplementary Table S3). This was consistent with other Australian evidence that suggested that discretionary foods tend to displace nutrient-dense core foods, including vegetables, in the diet [50,51]. The absence of explicit consideration of these types of foods in many sustainable diet studies and recommendations; e.g., [26], is a major concern that needs urgent attention. Additionally, the HQLI subgroup demonstrated that major environmental gains are possible without the need for Australians to exclude particular nutrient-dense core foods or food groups or adopt unfamiliar dietary habits. Compared to the overall adult population, the HQLI subgroup had lower results in every environmental impact category (Table 1) and less than half of the climate impact. Importantly, the dietary habits of the HQLI subgroup could be adopted by all Australian adults immediately and without the dietary risks associated with limiting or excluding any of the nutrient-dense core food groups (Table 2) [39,40,41,42,43,44].

### 4.2. Addressing Barriers to Higher Vegetable Intake

Daily diets were selected for the HQLI subgroup based on a higher overall diet quality score, and vegetable intake varied greatly between the individual diets. Most daily diets did not achieve the *Australian Dietary Guidelines’* recommended intake for vegetables (a minimum of five or six servings depending on the specific age group and gender), although about one quarter of the subgroup did (around 24%; Table 3). A higher vegetable intake was associated with a greater variety of vegetable intake, consistent with other studies [65,66,67]. A higher vegetable intake was also associated with a lower intake of bread and cereal foods, as well as a higher intake of red meat (Table 4). These results also were consistent with other evidence. For example, around a decade ago, Jenkins et al. [68] studied more than 10,000 diets obtained from the Australian Longitudinal Study on Women’s Health, and reported that higher intakes of unprocessed red meat, chicken, and fish were all associated with higher intakes of vegetables. More recently, Dogbe and Revooredo-Giha [69] demonstrated that fiscal policies designed to increase fruit and vegetable intake in the UK would also increase the intake of eggs and fresh meats, including beef, veal, lamb, and pork. This study was based on economic modeling of food choices and substitutions under changing fruit and vegetable prices. Contrasting results were reported by Colombo et al. [70], but in this case, the modeling explored a hypothetical dietary scenario whereby increases in vegetable consumption were substituted for meat. In other words, this was an a priori modeling decision by the researchers. Our finding that adult diets in Australia with a higher vegetable intake and lower environmental impacts had more red meat and fewer grains supported the view that animal- and plant-sourced nutrition should be viewed as complementary, not competitive [71]. These results underscored the importance of examining the complete diet, including the local context that determines the interrelationships between the various types of food eaten in meal combinations. It was also important that the diets in the HQLI subgroup with a higher vegetable intake also had a greater likelihood of achieving the estimated average requirements for several individual nutrients (Table 5), particularly those nutrients that are most often underconsumed by Australian adults. The higher vegetable intake tertile was also associated with a higher intake of discretionary foods (2.55 compared to 2.15 servings). However, as noted already, this was far below the population estimate of 6.8 servings and was not of great concern, given that it fell within the recommendations for health.

Many barriers to increasing the intake of vegetables in the diet have been identified. To begin, surveys in Australia have found poor awareness that vegetable intake is inadequate, especially among males [72]. A lack of interest in and understanding of maintaining good health has also been linked to low fruit and vegetable consumption [73]. Beyond knowledge and motivation, cost has also been identified as a factor [74,75,76], along with availability in some rural and remote areas [77]. Whatever the underlying reasons are, the evidence suggested that lower-than-recommended vegetable intake across the Australian population is a long-term issue [2]. The *Australian Dietary Guidelines* [23] recommend that Australians “…enjoy plenty of vegetables, including different types and colours and legumes every day”. Greater emphasis on strategies to increase variety is warranted as a way to increase vegetable consumption. Other strategies could include meal planning as a potential way of supporting higher vegetable intake. Vegetables are seldom eaten alone. Therefore, to encourage greater vegetable intake, there is a need to encourage the types of meals that include substantial quantities of vegetables. This could be in the form of recipe guides, weekly meal planners, or even the use of home-delivered semiprepared meal kits, which have become widely available in Australia in recent years. In addition, there is a need to encourage the number of eating occasions when vegetables are included. In Australia, vegetables are predominantly consumed with dinner [78,79]. Vegetable intake could be increased if the types of meals containing vegetables were also consumed more frequently for lunch and at other times during the day. Further investigations of meal types and eating occasions would be valuable.

### 4.3. Limitations

This study used Australian dietary intake data and environmental data applicable to foods in the Australian food system. As such, the findings reported were specific to Australia, and may not be relevant to other regions where food culture differs, and where systems of food production and environmental challenges differ. In every respect, efforts were taken to use the highest-quality data available. The dietary-intake data came from the Australian Health Survey, which is a large, nationally representative survey undertaken by the Australian Bureau of Statistics. These data are considered to be of high quality and reliable, as they form the basis for public health nutrition policy in Australia. Nevertheless, the 24-h dietary-recall process is subject to under-reporting, and although estimates of under-reporting prevalence were applied, these factors were applied uniformly across the data, meaning that it was possible that vegetable intake was marginally overestimated and discretionary food intake was marginally underestimated. This was due to the potential for under-reporting bias toward discretionary foods [80]. The study used an environmental impact score based on four environmental indicators. As such, not every environmental aspect was included. However, four indicators was a reasonable number, and provided a much more reliable environmental assessment than a single indicator, such as GHG emissions or water use alone. The study did not include an assessment of food waste due to a lack of sufficient data at the individual food level. Food waste is considered a major environmental issue [81]. However, exclusion of food waste was unlikely to have a bearing on the study’s conclusions. Similarly, food packaging was not considered, because the dietary intake survey did not collect this information and, in Australia, the same foods can be packaged in a variety of formats. Again, this exclusion was unlikely to have any bearing on the study’s conclusions.

## 5. Conclusions

Dietary patterns were prevalent in the Australian community that had higher levels of vegetable intake and substantially lower environmental impacts. These diets were characterized by higher levels of variety in the types of vegetables eaten, lower levels of intake of bread and cereal foods, and a higher intake of red meats. These diets also had a greater likelihood of achieving the recommended intake of a variety of vitamins and minerals. These results underscored the importance of considering the total diet when developing strategies to promote healthy and sustainable food consumption, rather than focusing on individual foods. These results also highlighted the importance of the inter-relationships between foods that exist in a local cultural context. Vegetables are seldom eaten on their own, and higher vegetable consumption in Australia could be supported by encouraging more regular consumption of the types of meals that include larger quantities of vegetables. This is possible while also substantially lowering total dietary environmental impacts.

## Figures and Tables

**Table 1:** Characteristics of the higher diet quality/lower environmental impact (HQLI) subgroup (*n* = 1700) compared to the population estimate (*n* = 9341).

Characteristic	HQLI Subgroup	Population Estimate	*p*-Value
Diet-quality score (out of 100)	59.2	42.6	<0.001
Climate footprint (kg CO_2_-e day^-1^)	1.55	3.33	<0.001
Water-scarcity footprint (L-e day^-1^)	301	394	<0.001
Cropland-scarcity footprint (m^2^y-e day^-1^)	4.90	6.89	<0.001
Pesticide-toxicity footprint (points day^-1^)	16.5	25.1	<0.001
BMI category (%)			0.091
Underweight	1.3	1.5	
Normal range	32.3	30.7	
Overweight	29.7	31.3	
Obese	20.8	21.9	
Dairy avoidance (%)	5.9	4.7	0.029
Activity level (past week) (%)			<0.001
Inactive	16.8	20.4	
Insufficiently active	28.3	26.4	
Sufficiently active	54.2	52.5	
Smoking status (%)			<0.001
Current daily smoker	9.8	15.8	
Current occasional smoker	0.9	1.9	
Ex-smoker	29.3	30.8	
Never smoked	60.1	51.6	
Level of highest education (%)			<0.001
Postgraduate	11.3	8.8	
Bachelor	19.3	18.2	
Certificate/Diploma	32.0	35.0	
Without post-school qualification	35.8	36.7	
SEIFA quintile (%) ^1^			0.186
Lowest 20%	17.6	17.9	
Second quintile	21.0	20.4	
Third quintile	20.5	20.0	
Fourth quintile	20.0	19.3	
Highest 20%	20.9	22.3	

^1^ Geographically determined socioeconomic index.

**Table 2:** Nutrient density of the higher diet quality/lower environmental impact (HQLI) subgroup (*n* = 1700) compared to the population estimate (*n* = 9341).

Nutrient	HQLI Subgroup	Population Estimate	Difference (%) ^1^
LCn3 (mg MJ^−1^)	52.1	34.6	50.5 **
Retinol equivalents (μg MJ^−1^)	134.0	100.8	32.9 **
Dietary fiber (g MJ^−1^)	3.6	2.7	30.7 **
Dietary folate equivalents (μg MJ^−1^)	91.6	74.4	23.2 **
Thiamin (B1) (mg MJ^−1^)	0.2	0.2	19.6 **
Magnesium (mg MJ^−1^)	47.4	40.5	17.1 **
Vitamin E (mg MJ^−1^)	1.4	1.2	15.9 **
Potassium (mg MJ^−1^)	395.7	345.7	14.5 **
Riboflavin (B2) (mg MJ^−1^)	0.3	0.2	14.4 **
Selenium (μg MJ^−1^)	12.3	10.8	13.9 **
Iron (mg MJ^−1^)	1.5	1.3	12.8**
Iodine (μg MJ^−1^)	23.3	20.8	12.0 **
Calcium (mg MJ^−1^)	107.5	96.1	11.9 **
Vitamin C (mg MJ^−1^)	14.0	12.5	11.9 **
Niacin (B3) equivalents (mg MJ^−1^)	5.5	4.9	11.9 **
Vitamin B6 (mg MJ^−1^)	0.2	0.2	9.7 **
Alpha-linolenic acid (g MJ^−1^)	0.2	0.2	8.9 **
Protein (g MJ^−1^)	11.6	10.7	7.7 **
Phosphorus (mg MJ^−1^)	185.7	172.8	7.5 **
Polyunsaturated fatty acids (g MJ^−1^)	1.4	1.3	6.9 **
Caffeine (mg MJ^−1^)	23.1	21.7	6.2 *
Linoleic acid (g MJ^−1^)	1.1	1.1	5.2 **
Total carbohydrates (g MJ^−1^)	27.5	26.2	5.0 **
Vitamin B12 (μg MJ^−1^)	0.5	0.5	1.5
Zinc (mg MJ^−1^)	1.3	1.3	−0.3
Sodium (mg MJ^−1^)	276.4	287.1	−3.7 **
Monounsaturated fatty acids (g MJ^−1^)	3.1	3.2	−3.8 **
Total fats (g MJ^−1^)	7.9	8.3	−5.6 **
Saturated fatty acids (g MJ^−1^)	2.7	3.1	−14.5 **
Trans-fatty acids (mg MJ^−1^)	126.1	156.1	−19.2 **
Free sugars (g MJ^−1^)	4.4	6.6	−32.4 **
Alcohol (g MJ^−1^)	0.6	1.6	−64.0 **

^1^ * *p* < 0.05; ** *p* < 0.01.

**Table 3:** The higher diet quality/lower environmental impact (HQLI) subgroup of adult (19 years old and above) daily diets in Australia: vegetable intake and variety score.

Group	Number	Servings/Day ^1^	Variety Score ^2^
Low vegetable intake tertile	550	0.29	1.6
Medium vegetable intake tertile	523	2.21	2.6
High vegetable intake tertile	627	7.53	3.3
Diets achieving recommended vegetable intake	412	8.91	3.4
Diets below recommended vegetable intake	1288	1.69	2.2
All HQLI diets	1700	3.34	2.5

^1^ A standard serving of vegetable is 75 g [23]. ^2^ Results obtained using alternative variety scores are reported in Supplementary Table S4.

**Table 4:** The higher diet quality/lower environmental impact (HQLI) subgroup of adult (19 years old and above) daily diets in Australia: food intake (servings person^−1^) for the low, medium, and high vegetable intake tertiles, for those diets achieving the *Australian Dietary Guidelines’* recommended intake for vegetables, and those diets below recommended vegetable intake ^1^.

Food Group	Servings per Person ^1^				
	Tertiles of Vegetable Intake			Recommended Vegetable Intake	
	Low	Medium	High	Achieved	Below
Fruit	1.61	1.64	1.59	1.52	1.64
Vegetables	0.29	2.21	7.53	8.91	1.69
Bread and cereal foods	5.60	5.11	4.32	4.37	5.20
Meats and alternatives	2.08	2.40	2.41	2.45	2.25
Fish	0.35	0.37	0.29	0.31	0.35
Beef and lamb	0.28	0.53	0.69	0.73	0.43
Poultry	0.77	0.76	0.62	0.60	0.75
Pork	0.08	0.20	0.19	0.18	0.15
Eggs, nuts, etc.	0.60	0.56	0.60	0.61	0.58
Reptiles, offal, etc.	<0.01	<0.01	0.02	0.03	<0.01
Dairy and alternatives	1.31	1.15	1.18	1.14	1.23
Discretionary choices	2.15	2.22	2.55	2.70	2.19

^1^ Food groups are as defined in the *Australian Dietary Guidelines* [23].

**Table 5:** The higher diet quality/lower environmental impact (HQLI) subgroup of adult (19 years old and above) daily diets in Australia: percent meeting nutrient estimated average requirements (EARs) for the low, medium, and high vegetable intake tertiles, for those diets achieving the *Australian Dietary Guidelines’* recommended intake for vegetables, and those diets below recommended vegetable intake ^1^.

Nutrient	Percent Meeting EAR ^1^				
	Tertiles of Vegetable Intake			Recommended Vegetable Intake	
	Low	Medium	High	Achieved	Below
Niacin (B3) ^2^	99.6	99.9	99.9	99.8	99.8
Phosphorus	96.7	99.2	99.5	99.5	98.2
Vitamin C	68.4	90.4	96.3	97.1	81.5
Protein	90.0	96.5	95.3	95.7	93.4
Folate ^3^	84.8	84.9	90.5	88.3	86.3
Iron	75.7	84.2	90.1	90.7	81.2
Riboflavin (B2)	79.0	83.1	85.0	85.4	81.5
Selenium	75.4	86.0	82.0	81.5	81.0
Magnesium	56.2	59.4	78.8	79.5	60.4
Thiamin (B1)	64.1	75.2	78.5	77.0	71.3
Vitamin B12	83.4	81.3	78.0	76.4	82.3
Iodine	84.3	82.5	77.7	76.7	82.9
Vitamin A ^4^	38.6	60.4	76.0	75.0	53.4
Vitamin B6	44.7	53.5	72.0	75.0	51.3
Zinc	43.3	56.0	67.9	68.5	51.9
Calcium	37.9	35.5	37.2	39.9	35.9

^1^ EARs are as defined by the nutrient reference values published by the National Health and Medical Research Council in Australia [61]. ^2^ Niacin equivalents. ^3^ Dietary folate equivalents. ^4^ Retinol equivalents.

## Data Availability

The dietary intake data are available from the Australian Bureau of Statistics (https://www.abs.gov.au/statistics/health/health-conditions-and-risks/national-health-survey-first-results/latest-release, accessed on 31 August 2017).

## Supplementary Materials

The following are available online at https://www.mdpi.com/article/10.3390/nu14071517/s1, Table S1: Weighting factors applied to footprint indicators to develop the environmental impact score, Table S2: Sample size for the higher diet quality and lower environmental impact (HQLI) subgroup according to age group and gender, Table S3: Food intake (servings/person/day) for the HQLI subgroup of adult diets, Table S4: HQLI subgroup of Australian adult daily diets: variety scores for vegetable intake.
